# Cultural values and mental health support seeking in older Nigerians living with HIV

**DOI:** 10.1093/geroni/igaf122.2675

**Published:** 2025-12-31

**Authors:** Candidus Nwakasi, Chinelo Nduka

**Affiliations:** University of Connecticut, Storrs, Connecticut, United States; Nnamdi Azikiwe Teaching Hospital, Nnewi, Anambra, Nigeria

## Abstract

**Background:**

About 2 million people are living with HIV (PLWHIV) in Nigeria. The prevalence rate is 2.3% for suicide attempt, 2.9% for suicide ideation, 7% for alcohol abuse, and 28.2% for depression in this group. To add to our understanding of this burden and how to mitigate it, this study examined the associations between cultural values, mental health seeking attitudes and intentions among older PLWHIV in Nigeria.

**Methods:**

We recruited 150 participants (Mage = 55.2) who identified as PLWHIV. They completed a questionnaire assessing sociocultural and sociodemographic factors, mental health seeking, and health outcomes measures. Multivariate linear regression models were fitted to determine if masculinity and collectivism were associated with mental health seeking attitude (MHSA), intention to seek mental health support from family during emotional/personal problems (MHSIF-E), and when having suicidal thoughts (MHSIF-S). Age, gender, education, relationship status, and perceived stress were covariates in the models.

**Results:**

Aging is associated with decreased MHSA (β = -0.172, p < 0.019), those who are partnered are more likely to report higher MHSA (β = 3.955, p < 0.005) and MHSIF-E (β = 2.775, p < 0.00), and higher perceived stress is associated MHSA (β = 0.334, p < 0.014). Increased masculinity (β = 0.417, p < 0.02) and collectivism (β = 0.251, p < 0.018) are associated with increased MHSA. Increased MHSA is associated with increased MHSIF-E (β = 0.203, p < 0.002) and MHSIF-S (β = 0.259, p < 0.001).

**Conclusion:**

To strengthen access and tailor HIV-related mental health interventions in Nigeria, programs must consider cultural values, mental health seeking attitudes, and intentions to seek support.

